# Energy expenditure and cellular activity underlie antibiotic tolerance of *Pseudomonas aeruginosa*

**DOI:** 10.1128/mbio.03968-25

**Published:** 2026-02-19

**Authors:** Michael F. Gates, Kim Lewis

**Affiliations:** 1 Antimicrobial Discovery Center, Department of Biology, Northeastern University 198849, Boston, Massachusetts, USA; University of California, Berkeley, Berkeley, California, USA

**Keywords:** antibiotic tolerance, persisters, *Pseudomonas aeruginosa*, bioenergetics

## Abstract

Persisters are a subpopulation of bacterial cells that survive a lethal dose of antibiotic. The failure to treat infections has been linked to the presence of these drug-tolerant cells. The recalcitrant, and often incurable, infection of cystic fibrosis airways by *Pseudomonas aeruginosa* is attributed to persisters found within aggregate biofilms that contain stationary cells. In all bacteria studied, the fraction of persisters is the highest in stationary populations. However, the level of persisters is unusually low in stationary phase *P. aeruginosa*, which is unexpected, given the recalcitrance to antibiotic therapy. Here, we set out to investigate the mechanism of *P. aeruginosa* antibiotic susceptibility in stationary phase. We find that *P. aeruginosa* is highly active in stationary phase, based on its rate of translation, and this correlates with increased killing compared to *Escherichia coli*. Growth within alginate beads improves *P. aeruginosa* stationary cell tolerance to antibiotic treatment, likely due to a subpopulation of low-translating cells. Secondary messenger cyclic diguanylate (c-di-GMP) regulates biofilm formation, and we find that a population of low-translating persisters also appeared in planktonically growing cells when c-di-GMP production is induced. This phenotype only occurred when exopolysaccharide production was intact, and we propose that the energetic demand of EPS overproduction induces dormancy. The level of energy expenditure appears to determine persistence in *P. aeruginosa. In vivo*-like growth conditions and a shift to a biofilm lifestyle, mediated by high c-di-GMP production, increase antibiotic tolerance by generating low-energy, dormant persisters.

**IMPORTANCE:**

Recalcitrant bacterial infections are a continued burden on the healthcare system. Antibiotic treatment failure, especially for chronic infections, can be attributed to persisters, a subpopulation of dormant cells that survive a lethal dose of drug. The infection of cystic fibrosis (CF) airways by *Pseudomonas aeruginosa* is often incurable to treatment by multiple classes of antibiotics due to the presence of persisters. *P. aeruginosa*, however, is highly susceptible to antibiotic killing *in vitro*, in apparent contradiction of its drug tolerance during infection. Here, we show that *P. aeruginosa* susceptibility to antibiotic killing is due to its continued protein synthesis and cellular activity even with entrance into stationary phase. Furthermore, we identify that the greater energetic demand of biofilm growth generates a larger fraction of low-translating persisters and increases antibiotic tolerance. These findings improve our understanding of *P. aeruginosa* antibiotic tolerance during CF infection and will aid the development of better treatment regimens.

## INTRODUCTION

Failure to treat bacterial infections is usually attributed to antibiotic resistance. Often, however, isolates from chronic infections are not resistant to the administered drugs and yet persist (1). In this context, antibiotic tolerance is the likely determining factor of therapeutic failure (2). Colonization and infection of the cystic fibrosis (CF) airways by *Pseudomonas aeruginosa* contributes to increased mortality (3), while lengthy antibiotic treatment of patients suffering from infection does not eradicate the pathogen (4). Isolates of *P. aeruginosa* from CF patients have been shown to generate a sub-population of tolerant persister cells that survive treatment and regrow when drug concentration decreases (5, 6). The molecular basis for the formation and survival of these *P. aeruginosa* persisters, especially in the context of infection, is not well understood.

The majority of what is known about antibiotic tolerance and persister formation has been obtained from the study of *Staphylococcus aureus*, *Salmonella enterica*, *Mycobacterium tuberculosis*, and *Escherichia coli. E. coli* is also the longest-studied model organism for understanding persisters. The *E. coli* toxin *hipA* was the first gene implicated in persister formation (7). This toxin phosphorylates glutamyl-tRNA-synthetase at its ATP-binding site, causing a buildup of uncharged tRNA^Glu^ and inhibiting translation, leading to dormancy (8). A mutant allele, *hipA7*, was selected for *in vitro*, and urinary tract infection isolates of *E. coli* were shown to carry mutant alleles of *hipA* conferring a high persister (hip) phenotype (9). Another toxin, TisB, was shown to be induced upon activation of the SOS response, whereby it forms an ion channel in the bacterial membrane, depleting proton motive force and ATP, causing dormancy (10–12). The low energy state caused by TisB foreshadowed work done first in *S. aureus* (13, 14), and subsequently in *E. coli* (15, 16), *Salmonella* Typhimurium (17, 18), *M. tuberculosis* (19), and *P. aeruginosa* (20), whereby low ATP was directly linked as an underlying general mechanism of persister formation—tolerance of antibiotics resulting from low activity of their targets. Single-cell analysis of *E. coli* persisters further confirmed that cells surviving treatment with β-lactams were low in ATP prior to drug being administered (21). The drop in ATP was shown to correlate with low expression of “noisy” key metabolic enzymes (21, 22), and overexpression of these enzymes quenched this “noise,” increasing ATP and improving susceptibility to antibiotics (19, 23). The role of ATP depletion has been disputed; however, instead, proposing that slow growth alone is sufficient for tolerance (24), and expression of the previously mentioned HipA toxin protects from killing, but cells still maintain high ATP (25). These observations should be addressed to understand how they are related to ATP depletion to better our understanding of antibiotic tolerance.

Stationary phase bacteria have greater drug tolerance than exponentially growing cells (26). Relative to other bacteria, *P. aeruginosa* stationary populations produce considerably less persister cells, and the bulk is highly sensitive to killing by fluoroquinolones (27) and aminoglycosides (28). This is paradoxical, as *P. aeruginosa* is known for its recalcitrance during infection of CF airways (29). *In vitro* evolution of tolerance in *P. aeruginosa* has identified respiratory mutants whose extended lag phase improves drug survival (4), and transposon sequencing (Tn-seq) has shown that loss of *de novo* pyrimidine synthesis sensitizes exponentially growing cells to treatment in an ATP-dependent manner (20).

Limitations exist when studying antibiotic tolerance *in vitro*. Other than *hipA7* (9), virtually no persister genes evolved through *in vitro* evolution experiments have been identified in clinical isolates. Mechanistic insight has been gained from studying tolerance development in clinical isolates themselves, with mutations in the phosphotransferase system identified in *E. coli* (16), and mutations in RNA polymerase subunit (*rpoC*), transcriptional repressor of purine biosynthesis (*purR*), or Clp protease subunit (*clpX*) conferring drug tolerance in *S. aureus* prior to the emergence of drug resistance (30). Furthermore, experiments with intracellular pathogens have shown the importance of the host environment on triggering persistence (31, 32). The CF airway presents a unique environment, with *P. aeruginosa* displaying an array of adaptations (33–35). *In vitro* models have now been designed to accurately replicate growth in the CF sputum, reproducing the conditions and metabolites available to *P. aeruginosa* in the lung (36, 37). Leveraging these models to better understand *P. aeruginosa* antibiotic tolerance will provide useful insights into how this bacterium persists during infection.

In this study, we assess *P. aeruginosa* antibiotic tolerance in stationary phase and within a CF alginate bead biofilm model, using the recently developed THRONCAT (38) to measure translation rate as a reporter of cellular activity. We first show that *P. aeruginosa* sensitivity to antibiotic in stationary phase is best predicted by the rate of translation. Furthermore, growth of *P. aeruginosa* in alginate beads generates a low-translating population and improves antibiotic tolerance. Increasing c-di-GMP levels in planktonically growing cells mimics the phenotype in beads and is dependent on the production of energetically costly exopolysaccharides (EPS). The approach described here outlines the interplay between energy expenditure and cellular activity in determining drug susceptibility.

## MATERIALS AND METHODS

### Bacterial strains and culture conditions

*P. aeruginosa* PAO1 and *E. coli* MG1655 were grown in Mueller-Hinton broth II (MHIIB; *P. aeruginosa* and *E. coli*) or cystic fibrosis sputum media (CFSM; *P. aeruginosa*) at 37°C, shaking at 225 rpm. The components of CFSM and their respective concentrations are listed in Table S1. When necessary for selection, gentamicin (60 μg/mL for *P. aeruginosa* and 10 μg/mL for *E. coli*) and carbenicillin (150 μg/mL for *P. aeruginosa* and 50 μg/mL for *E. coli*) were added. For the induction of the P_BAD_ promoter, arabinose was added to media at a final concentration of 0.2%.

Gene deletions were constructed using two-step allelic exchange (39)—for each target gene, pEX18Gm was first digested using SmaI, and then ~500 bp of homology from both up and downstream (~1,000 bp total) of the target gene was inserted into vector pEX18Gm using Gibson Assembly (40). pEX18Gm was conjugated by puddle mating using *E. coli* SM10(λpir) into PAO1 and selected on pseudomonas isolation agar containing gentamicin (60 μg/mL). Counterselection on no-salt lysogeny broth containing 15% sucrose was immediately followed by verification of the gene deletion by PCR and loss of gentamicin resistance.

PAO1 Δ*pvdD* was a gift from Samuel Miller’s lab at the University of Washington, Seattle, as were the pTn7 vectors harboring the arabinose-inducible diguanylate cyclase (DGC CC3285) or phosphodiesterase (PDE CC3396) from *Caulobacter crescentus* (41). The Tn7 transposon containing the DGC or PDE was introduced either by triparental mating or simultaneous electroporation of the pTn7-ara-DGC or pTn7-ara-PDE with vector pTN52 containing the necessary transposase to facilitate transposition (42). Insertions into the attTn7 site downstream of the *glmS* gene were verified by PCR using primers specified by Choi et al. (42).

PAO1 Δ*pelA* Δ*pslBCD* Δ*algD* (designated ΔEPS) was a gift from Tim Tolker-Nielsen and Thomas Bjarnsholt at the University of Copenhagen, Denmark.

### Antibiotic susceptibility assays

The minimum inhibitory concentration (MIC) was determined using broth microdilution. MICs were determined in both MHIIB and CFSM. MICs of *P. aeruginosa* grown in alginate beads were determined by placing individual beads containing 10^5^ CFU/mL of *P. aeruginosa* into 96-well plates prepared with antibiotic by microdilution. CFU was determined before and after incubation with antibiotic for 20–24 h. The minimal bactericidal concentration was simultaneously determined by plating CFU as well.

Antibiotic tolerance was determined by time-kill assays. First, a seed culture was grown overnight (~16 h) in MHIIB. Planktonic killing experiments in MHIIB were done in 125 mL Erlenmeyer flasks containing 25 mL of media. Overnight cultures were diluted 1,000-fold and grown again overnight (24 h) to stationary phase. Initial CFU were determined by serial dilution and plating on MHIIB agar. Cultures were then challenged with 10× MIC of antibiotic (for *P. aeruginosa*, ciprofloxacin at 1.5 μg/mL and gentamicin 15.6 μg/mL; for *E. coli*, ciprofloxacin 0.4 μg/mL and gentamicin at 7.8 μg/mL). Cells were washed once with phosphate-buffered saline (PBS) prior to serial dilution and plating. CFU was determined at 2, 4, 6, and 24 h following the start of treatment. Time-kill assays in CFSM and in alginate beads were done as mentioned above but with several alterations. Overnight seed cultures were diluted into 5 mL of media or 2% alginate at a final OD600 of 0.01. The alginate beads were suspended in 5 mL of CFSM, and both the liquid cultures and suspended beads were grown overnight (24 h) to stationary phase in 50 mL Falcon tubes. The CFSM that alginate beads were suspended in was aliquoted and centrifuged at 8,000 × g for 5 min to pellet any planktonically growing cells. Beads were then resuspended in this spent media supernatant. Cultures were challenged with 10× MIC ciprofloxacin (PAO1 at 10 μg/mL). Cells were washed once in dissolving solution (DS; see below), serially diluted, and plated for CFU. See below for alginate bead preparation and method of CFU enumeration from alginate beads.

### Alginate bead preparation and viable cell counts

Alginate beads were prepared as described by Sønderholm and colleagues (36). To elaborate, alginate granules were sterilized by autoclave prior to encapsulation. A 2% alginate slurry was made by dissolving alginate in a sterile solution of 1% NaCl. A 50 mL beaker containing a stir bar was placed on a stir plate adjacent to a syringe pump and filled with 40 mL of a 0.25 M CaCl_2_ solution then stirred gently. After diluting *P. aeruginosa* into 5 mL of 2% alginate slurry (final OD600 0.01), this mixture was taken up by a syringe with a 21-gauge needle and set into the syringe pump. The needle was placed ~2 cm above the surface of the stirring CaCl_2_ solution, and the pump was engaged (0.5 mL/min rate). After completion, the syringe was removed, and the alginate beads were allowed to incubate while still stirring in the CaCl_2_ solution for an additional 15–20 min to fully harden. The CaCl_2_ was then removed, and the beads were washed with 1% NaCl and then resuspended in CFSM.

To determine CFU of *P. aeruginosa* grown in alginate beads, beads were first dissolved using a solution of Na_2_CO_3_ and citric acid as previously described (36). This DS was prepared fresh prior to enumeration by diluting a 1M stock of each component to a final concentration of 0.05 M Na_2_CO_3_ and 0.02 M citric acid. A single bead was suspended in a microcentrifuge tube containing 200 μL of DS and placed in a vortex mixer with tube adapters and vortexed for 15 min. The cell suspension was then serially diluted and plated on LB agar plates for CFU enumeration.

### βES incorporation assay

The threonine analog β**-**ethnylserine (βES) was generously provided by Kim Bonger at Radboud University. THRONCAT, as described by Bonger and colleagues (34, 38), was conducted as follows: a 200 mM stock of βES was thawed prior to the experiment. For incorporation in MHIIB, both *P. aeruginosa* and *E. coli* were back diluted 1,000-fold day-of and grown to OD600 0.3–0.4. A 1 mL aliquot was taken, and βES was spiked in at a final concentration of 4 mM for 1 h; a 1 mL no βES control was additionally aliquoted. After 1 h, cells were washed once with PBS and then fixed with 4% formaldehyde for 15 min. Cells were then washed twice with 3% bovine serum albumin (BSA), resuspended in PBS, and stored at −20°C. Cultures were allowed to reach stationary phase (24 h), and, again, 1 mL aliquots were again taken, and βES was spiked in at a final concentration of 4 mM for 1 and 4 h. A no βES control was included. At both 1 and 4 h, cells were prepared as described above and again stored at −20°C until further proceeding.

To further prepare samples for flow cytometry, samples were first thawed at room temperature, pelleted, and resuspended in 0.5% Triton X-100 and incubated at room temperature for 20 min. Cells were again washed twice with 3% BSA. The Invitrogen Click-IT HPG Kit was used to prepare a reaction cocktail as per the manufacturer’s instructions (43) with the modification that 5-TAMRA-azide (Jena Biosciences) was used as the clickable fluorophore at a final concentration of 2 μM. Cells were resuspended in 100 μL of the reaction cocktail and incubated in the dark at room temperature for 30 min. Cells were pelleted and washed in the reaction rinse buffer (component F from the Invitrogen Click-IT HPG Kit) once and then resuspended in a final volume of 100 μL PBS. These cell suspensions were diluted again in PBS and analyzed on a BD FACSAria II. Both forward and side scatter values were collected and used to gate populations by size. Fluorescence of TAMRA was collected in the PE channel. For all samples run, 100,000 events total were collected.

For βES incorporation in CFSM and in alginate beads, the above was done with additional steps to prepare the beads. At the time βES was to be added, the CFSM that the alginate beads were suspended in was aliquoted and centrifuged at 8,000 × *g* for 5 min to pellet any planktonically growing cells. A 1 mL aliquot was taken, 10 beads were added to this aliquot, and βES was added to a final concentration of 4 mM and incubated for 1 and 4 h. A total of 10 beads were dissolved to provide enough cells for exponential incorporation, while five beads were used for each timepoint during stationary incorporation. The planktonically grown cells in CFSM were assayed exactly as described in MHIIB.

### Motility assay

Determination of swimming motility was done as previously described (44). In short, a 5× M8 solution was prepared by dissolving 32 g Na_2_HPO_4_•7H_2_O, 7.5 g KH_2_PO_4_, and 1.25 g NaCl in 500 mL of deionized water. The final media included 1× M8 salts, 0.2% glucose, 0.5% casamino acids, 1 mM MgSO4, and 0.3% agar. Thick 25 mL plates were poured with the 0.3% agar M8 media. Strains of *P. aeruginosa* whose motility was being assayed were grown overnight in MHIIB. An aliquot of the overnight culture was taken, and the very tip of a 200 μL pipette tip was dipped into the culture then stabbed into the center of the M8 plate. These plates were then incubated upright at 37°C for 16–20 h.

### Microscopy

For microscopy of alginate beads, *P. aeruginosa* PAO1-iGFP was cultured in MHIIB overnight to then embedded into alginate beads (as previously described) and grown in CFSM at 37°C shaking. At 4 h and 24 h of incubation, a bead was removed, sliced in half, then again down the center to create a thin cross-section. Cross-sections were mounted on a microscope slide between cover glass and imaged on a Nikon Ti2-E fluorescence microscope using a 40× or 60× objective. Fluorescence signal for GFP was collected after excitation at 488 nm, as was a phase contrast image. Images were acquired with NIS-Elements at a resolution of 2,048 by 2,048 pixels.

## RESULTS

### *P. aeruginosa* antibiotic sensitivity is correlated with increased translation in stationary phase

**Fig 1:**
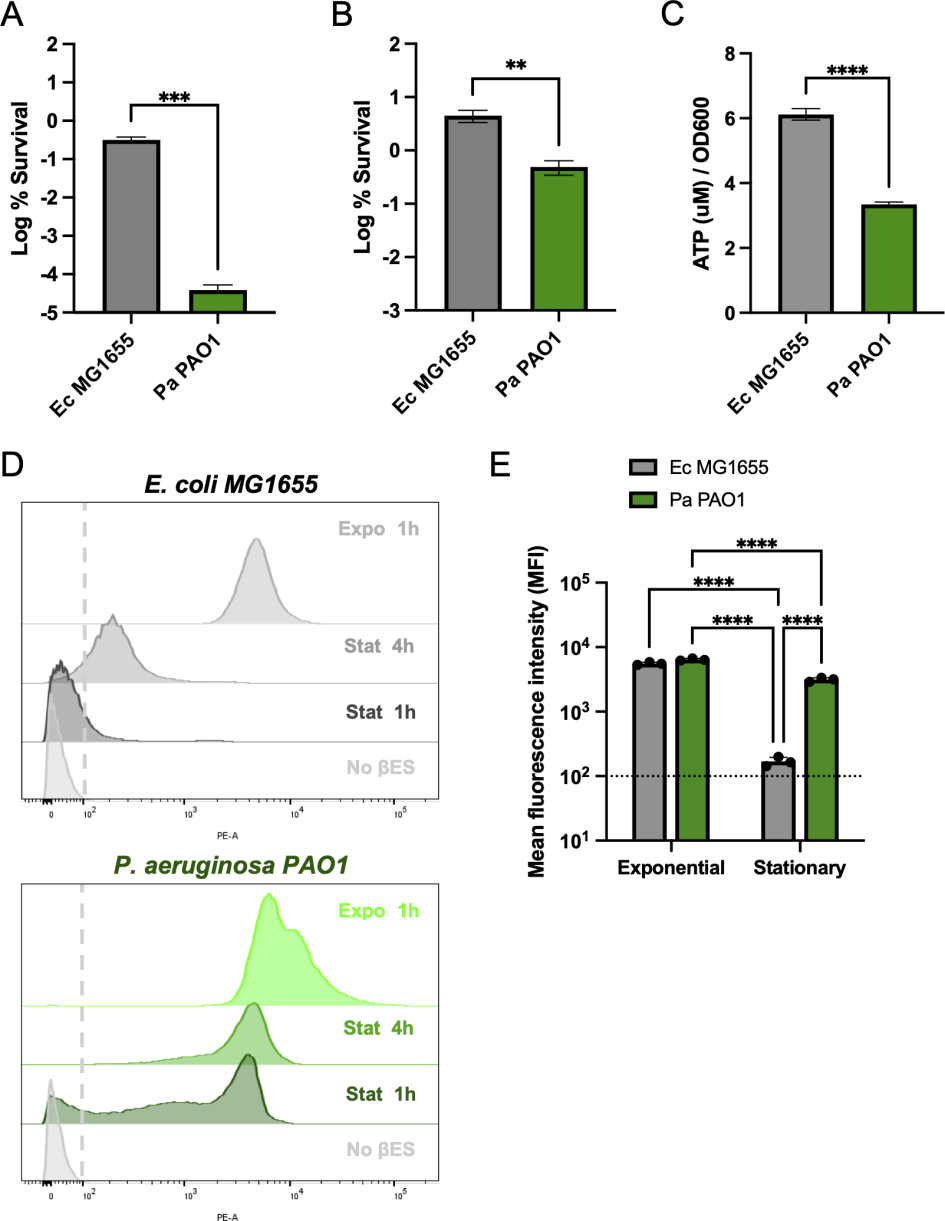
*P. aeruginosa* has increased antibiotic susceptibility and translation rate in stationary phase. Log % survival of stationary cultures after 24 h of treatment with 10× MIC of (**A**) ciprofloxacin and (**B**) gentamicin. (**C**) Bulk ATP measurements of stationary cultures. (**D**) THRONCAT—βES incorporation assay to measure translation in exponential and stationary cultures of *E. coli* MG1655 (gray) and *P. aeruginosa* PAO1 Δ*pvdD* (green). A dashed line indicates non-fluorescence cutoff. Each distribution is accompanied by the growth phase + time (**H**) incubated with βES; data collected on the FACS. (**E**) Mean fluorescent intensity values for exponential and stationary 1 h βES populations, dashed line is nonfluorescent cutoff. Data are representative of at least three biological replicates. Asterisks denote statistical significance as determined by either unpaired two-tailed *T* test (**A–C**) or one-way analysis of variance (ANOVA) followed by Tukey’s multiple comparisons test (**E**). **, *P* < 0.01; ***, *P* < 0.001; and ****, *P* < 0.0001.

To examine the antibiotic tolerance of *P. aeruginosa* in stationary phase, and to benchmark its sensitivity to antibiotic, we conducted time-kill assays on both *P. aeruginosa* PAO1 and *E. coli* MG1655. We grew bacteria planktonically in MHIIB to stationary phase and challenged them with either the fluoroquinolone ciprofloxacin or aminoglycoside gentamicin at 10× MIC. PAO1 exhibited significant sensitivity to ciprofloxacin in stationary phase, with a 6-log reduction in survival after 24 h (Fig. 1A) and a 2-log reduction with gentamicin (Fig. 1B). Relative to PAO1, *E. coli* displayed a 6,897-fold greater survival with ciprofloxacin (Fig. 1A), and a significant ninefold increase in the presence of gentamicin (Fig. 1B) after 24 h of treatment, indicating the heightened sensitivity to antibiotics by *P. aeruginosa*.

Previously, we suggested that a reduction in cellular energy, namely ATP, represents a general mechanism of persister formation (13, 15, 19, 20). As energy is depleted, the activity of antibiotic targets decreases and provides protection from killing. We quantified ATP of *E. coli* and *P. aeruginosa* in stationary phase and surprisingly found that *E. coli* had a greater level of ATP than PAO1 (Fig. 1C). We hypothesize that the lower ATP of *P. aeruginosa* could be the result of high energy expenditure through increased activity. Ultimately, bactericidal activity of antibiotics will be determined by the activity of targets, which means the rate at which they consume ATP, rather than the level of ATP *per se*. As a proxy for the general activity of a cell, we decided to use translation, where an estimated ~50% of all energy is consumed (45). We measured translation using THRONCAT (38), an incorporation method using the sensitive non-canonical amino acid βES. βES incorporation allows for a clickable fluorophore to be added to nascent proteins (38) and for measurements of translation via flow cytometry. *P. aeruginosa* encodes a nonribosomal peptide synthetase pyoverdine synthetase D (*pvdD*), which incorporates two L-threonine residues during synthesis of pyoverdine. To exclude the incorporation of the threonine analog βES into pyoverdine, we conducted THRONCAT using a Δ*pvdD* strain of PAO1. Incorporation of βES during exponential growth for 1 h was comparable between *E. coli* and PAO1 Δ*pvdD*, with mean fluorescence intensities (MFI) of 5,300 and 6,687, respectively, a slight ~1.2-fold difference in MFI (Fig. 1D and E). Stationary phase incorporation, however, was dramatically different. After 1 h of incubation with βES, 81% of the PAO1 population was fluorescence positive, whereas only 9.84% of the *E. coli* population was fluorescent and actively translating (Fig. 1D). The MFI for stationary PAO1 was 3,167, 22-fold higher than *E. coli* at 143.3 (Fig. 1E). In addition, a majority of this *E. coli* population overlapped with the no βES (nonfluorescent) control, indicating no detectable translation (Fig. 1D). Prolonged incubation for 4 h with βES resulted in overall fluorescence increase for both species; however, *E. coli* translation (MFI 315.7) remained 12-fold less than *P. aeruginosa* (MFI 3861). Strikingly, a large portion of stationary PAO1 cells had translation rates equivalent to exponential levels, as shown with the overlapping distributions on the far right (Fig. 1D), indicating that *P. aeruginosa* is highly active even in stationary phase.

### Alginate bead biofilm model increases antibiotic tolerance of *P. aeruginosa*

**Fig 2:**
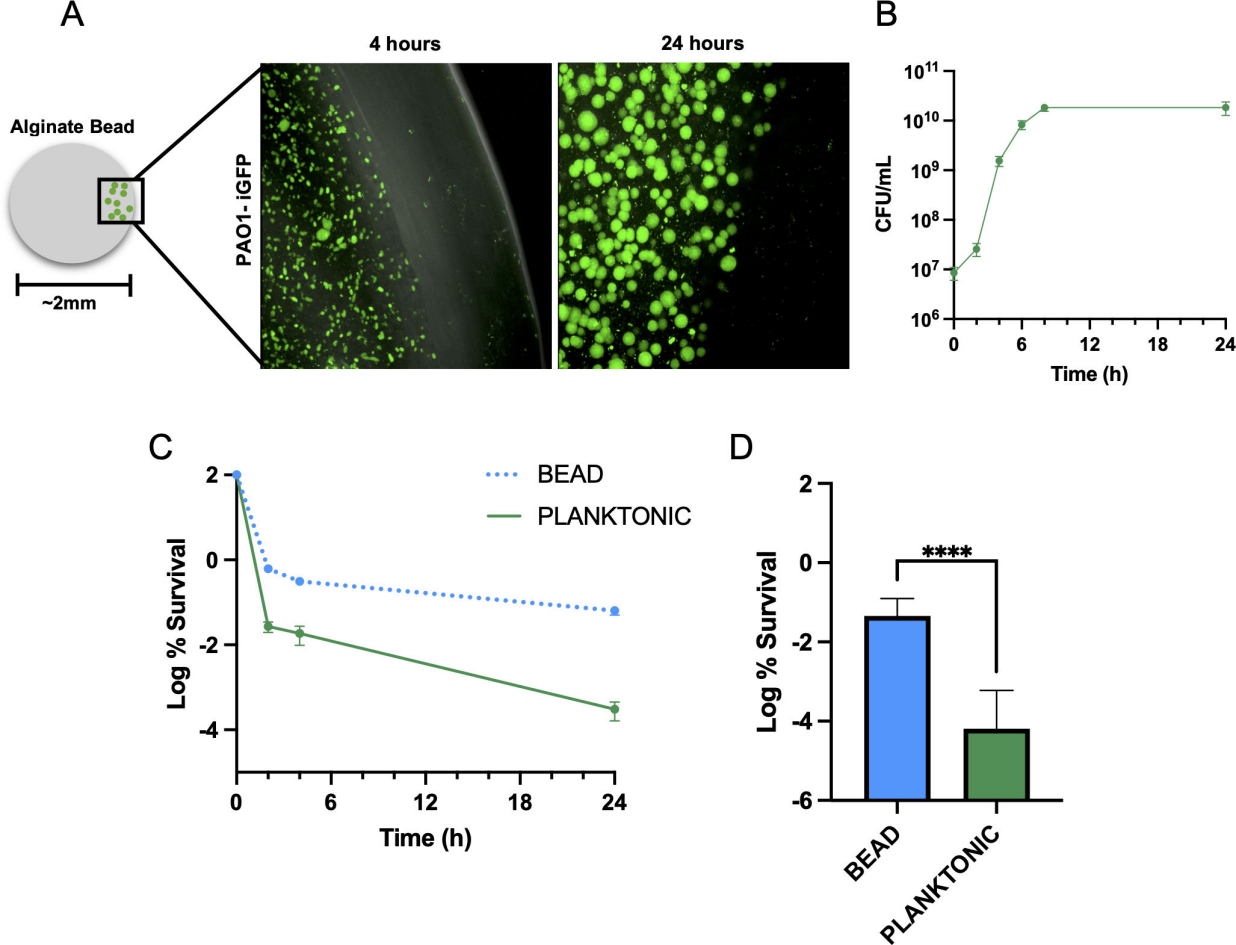
Alginate bead biofilm model induces antibiotic tolerance. (**A**) Schematic representation of an alginate bead and fluorescence microscopy images of *P. aeruginosa* PAO1-iGFP grown within the bead after 4 h and 24 h of growth. (**B**) Enumeration by CFU of alginate bead-embedded PAO1 grown in CFSM. (**C**) Time-kill kinetics of PAO1 grown planktonically and within alginate beads to stationary phase in CFSM, treated with 10× MIC ciprofloxacin. (**D**) Log % survival after 24 h treatment. Data are representative of at least three biological replicates. Asterisks denote statistical significance as determined by an unpaired two-tailed *t* test. ****, *P* < 0.0001.

*P. aeruginosa* is known for its recalcitrance and antibiotic tolerance, while in the CF lung, often seen in an aggregate biofilm state. We, therefore, adopted an *in vivo*-like alginate bead biofilm model (36) (Fig. 2A) to test antibiotic susceptibility. Encapsulation of *P. aeruginosa* PAO1 within the alginate bead resulted in sizable aggregates formed in 24 h (Fig. 2A). To better recapitulate the nutrients available within the CF lung, PAO1 within the alginate beads was grown in CFSM (Table S1), which supported robust growth (Fig. 2B). The MICs for ciprofloxacin and tobramycin were determined in beads and in CFSM. The MIC for ciprofloxacin was unchanged by growth in alginate bead (Table S2), while the MIC was increased fivefold in CFSM (Table S2). Tobramycin, however, showed a greater than 30-fold increase in MIC when assaying PAO1 in CFSM and a further twofold increase by the alginate bead (Table S2). We, therefore, focused on using ciprofloxacin to assay antibiotic tolerance, as it was less affected by the growth conditions. We grew PAO1 to stationary phase (24 h) both planktonically and within the alginate beads and challenged it with ciprofloxacin in CFSM (Fig. 2C). Planktonic PAO1 again showed a high level of sensitivity to ciprofloxacin in stationary phase, with a 5-log reduction in CFU over 24 h (Fig. 2C and D). Notably, a 240-fold increase in survival was seen over 24 h of treatment when PAO1 was grown in the beads (Fig. 2C and D), suggesting aggregate growth in the bead induces drug tolerance. Additionally, we tested whether this change in survival was due to limited growth in the interior of the alginate bead. Nitrate was incorporated into bead production, which has been shown to support robust growth throughout the bead (36). No difference in survival was seen with nitrate supplementation in stationary phase (Fig. S1).

**Fig 3:**
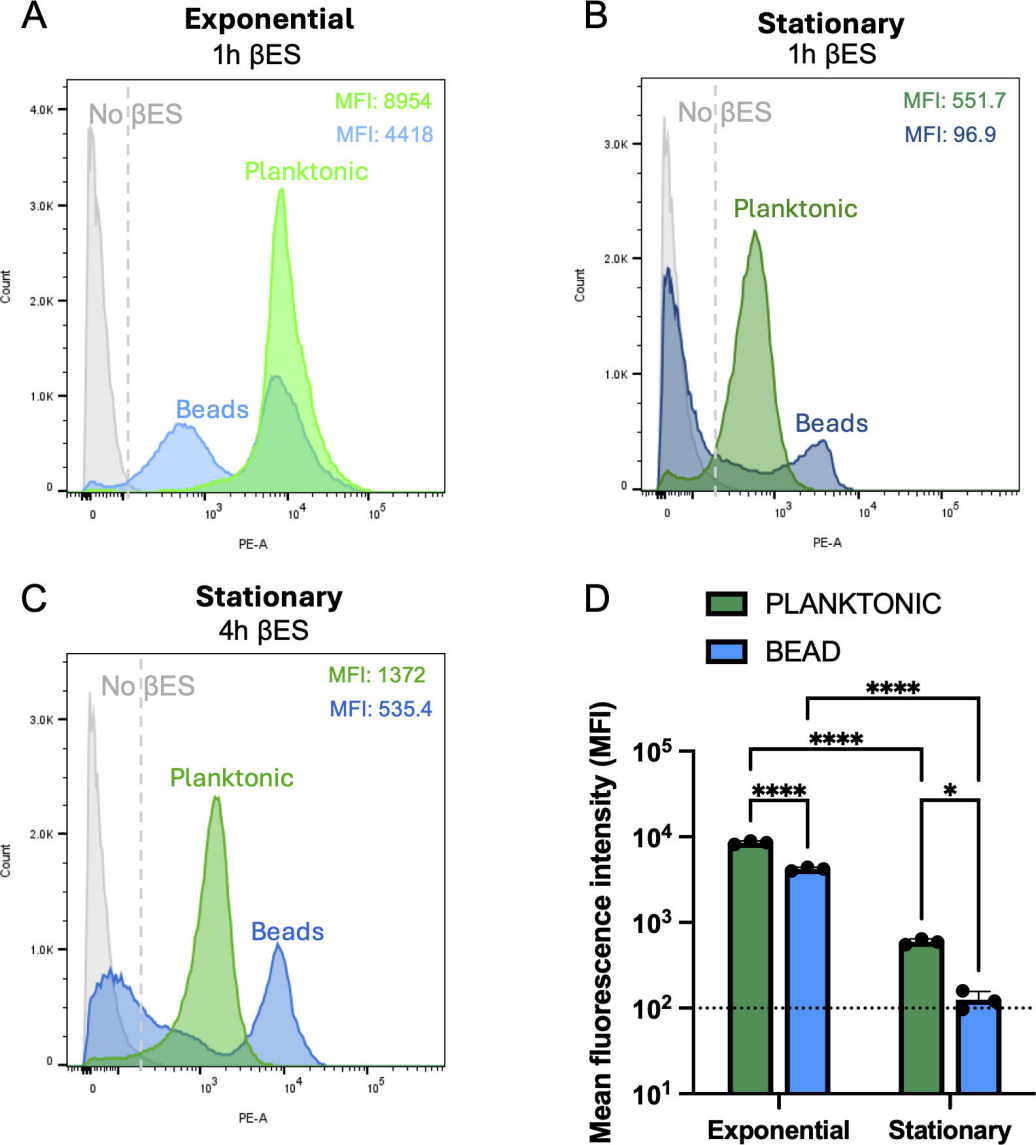
A population of low-translating cells exists during alginate bead growth. THRONCAT—βES incorporation assay to measure translation of *P. aeruginosa* PAO1 Δ*pvdD* grown planktonically (green) or within alginate beads (blue) during (**A**) exponential growth with βES for 1 h, (**B**) stationary phase with βES for 1 h, and (**C**) stationary phase with βES for 4 h. (**D**) Mean fluorescent intensity values for exponential and stationary 1 h βES populations from panels **A and B**, error bars are % CV, and dashed line is nonfluorescent cutoff. All data collected on the FACS. Data are representative of at least three biological replicates. Asterisks denote statistical significance as determined by one-way analysis of variance (ANOVA) followed by Tukey’s multiple comparisons test (**D**). *, *P* < 0.05; ****, *P* < 0.0001.

Having improved drug tolerance of PAO1 through growth in the alginate bead, we asked how the activity of this population had altered. We compared planktonic cells with those grown in alginate beads and assayed the percentage of the cell population undergoing active translation using THRONCAT (38). Exponential growth in the alginate bead (MFI 4418), interestingly, displayed a bimodal distribution, with one population overlapping with planktonically grown PAO1 (MFI 8954) and the other peak shifted left, showing decreased translation (Fig. 3A). The translation rate in stationary phase was different between the two growth conditions. A typical normal distribution is seen in planktonic cells with a reduced MFI of 551.7 compared to exponential growth (Fig. 3B and D). Additionally, a small shoulder of the distribution is shifted left of our no βES (nonfluorescent) control, indicating no translation (Fig. 3B). The alginate bead population in stationary phase is still bimodal; however, the vast majority of the population is now shifted left and overlaid with the no βES control, indicating no translation (MFI 96.9; Fig. 3B and D). Continued incubation with βES for 4 h showed further incorporation and increase in fluorescence for both planktonic (MFI 1372) and bead (MFI 525.4) populations, indicating that active translation is being measured and is occurring irrespective of growth (Fig. 3C), as CFU are stable after 8 h (Fig. 2B). The presence of more dormant, low-translating cells during alginate bead growth correlates with the increased survival we saw during antibiotic treatment.

### Prolonged high c-di-GMP increases survival in planktonic cells and decreases translation in stationary phase

**Fig 4:**
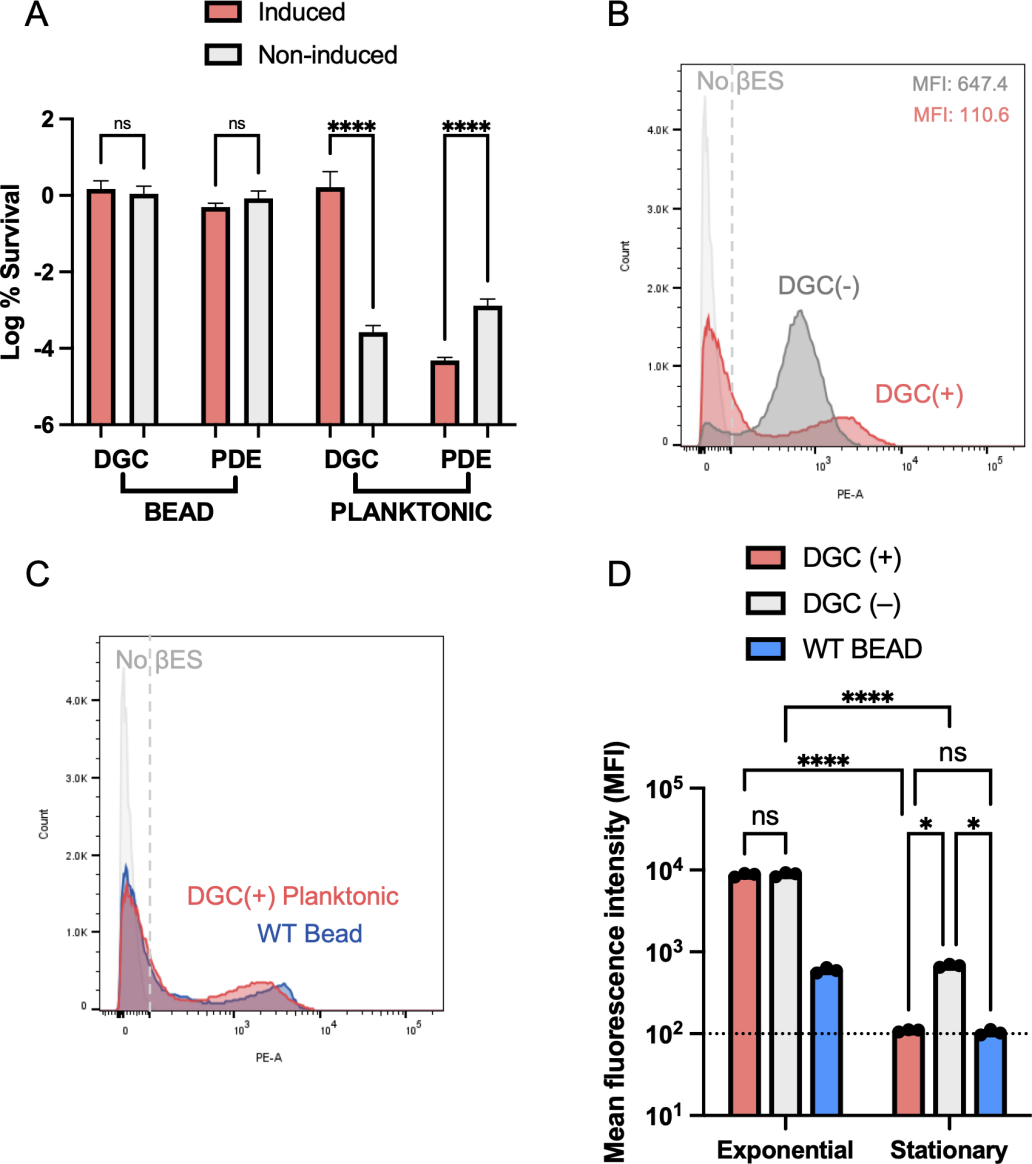
High c-di-GMP improves antibiotic tolerance of planktonic cells and generates a population of low-translating cells. (**A**) Twenty-four hour log % survival of 10× MIC ciprofloxacin-treated PAO1 P_BAD_-DGC or PAO1 P_BAD_-PDE grown planktonically or within alginate beads (24 h), with and without induction. (**B and C**) One hour βES incorporation assay to measure translation of PAO1 Δ*pvdD* P_BAD_-DGC, with (red) and without (gray) induction in stationary phase. (**C**) Comparison of translation rate in stationary phase of planktonically grown PAO1 Δ*pvdD* P_BAD_-DGC with induction vs. WT PAO1Δ*pvdD* grown in an alginate bead. (**D**) Mean fluorescent intensity values for exponential and stationary 1 h βES populations, dashed line is nonfluorescent cutoff. Asterisks denote statistical significance as determined by either two-way analysis of variance (ANOVA) (**A**) or one-way ANOVA (**D**) followed by Tukey’s multiple comparisons test. ns, *P* > 0.05; *, *P* < 0.05; ****, *P* < 0.0001.

The transition between free-swimming planktonic growth and biofilm formation in *P. aeruginosa* is regulated by c-di-GMP. High levels of c-di-GMP produced by DGCs induce biofilm formation and inhibit motility, whereas low levels of c-di-GMP, through hydrolysis by PDEs, stimulate motility and repress biofilm formation (46). To test whether the formation of aggregates within the alginate bead model (36) (Fig. 2A) and the improved survival to antibiotic challenge (Fig. 2C and D) were dependent on c-di-GMP-mediated biofilm formation, we utilized an inducible DGC and PDE to shift intracellular concentrations of c-di-GMP. DGC induction reduced motility (Fig. S2A) and caused clumping during growth in liquid (data not shown). No change in motility was seen for PDE induction (Fig. S2A). PAO1 containing P_BAD_-DGC or P_BAD_-PDE was grown shaking in CFSM to stationary phase (24 h) with and without inducer (0.2% arabinose). These stationary cultures were then challenged with 10× MIC ciprofloxacin and assayed for survival. Induction of the DGC or PDE in the alginate bead model had no effect on the antibiotic survival of PAO1 after 24 h (Fig. 4A). DGC induction and increase in c-di-GMP did, however, greatly improve survival during planktonic growth, with a 7,666-fold increase relative to the non-induced control (Fig. 4A). This level of survival is equivalent to that seen in alginate beads. The induction of PDE showed the inverse, sensitizing the planktonic culture to antibiotic by 28-fold (Fig. 4A).

To examine whether the dramatic survival improvement of planktonic cells with high c-di-GMP (DGC+; Fig. 4A) was due to alterations in translation, we again measured translation by THRONCAT. PAO1 Δ*pvdD* P_BAD_-DGC was grown with (DGC+) and without (DGC−) inducer planktonically and incubated with βES in the exponential and stationary phases. Exponential translation rates between induced DGC+ (MFI 8234) and non-induced DGC− (MFI 9268) were comparable, with a small subpopulation of low-translating cells detected in the DGC+ population (Fig. S2B). Stationary growth showed a bimodal distribution for DGC+, with a large peak situated below the no βES (nonfluorescent) control, again indicating no detectable translation, and a smaller peak representing an actively translating subpopulation (Fig. 4B). The non-induced (DGC−) population was evenly distributed with a small subpopulation of non-translating cells and a greater overall MFI of 647.4 compared to the induced (DGC+) MFI of 110.5 (Fig. 4B). Strikingly, when superimposing the bimodal distribution displayed by DGC induction in planktonic cells with that of PAO1 Δ*pvdD* grown in the alginate beads (Fig. 4C), both have identical distributions with similar MFI (Fig. 4D). Both exhibit a large population of non-translating cells (Fig. 4C) alongside their improved antibiotic tolerance (Fig. 2D and 4A), further indicating a connection between the emergence of this population and decreased susceptibility.

### Energy consumption through EPS production is necessary for increased survival of high c-di-GMP producing cells

**Fig 5:**
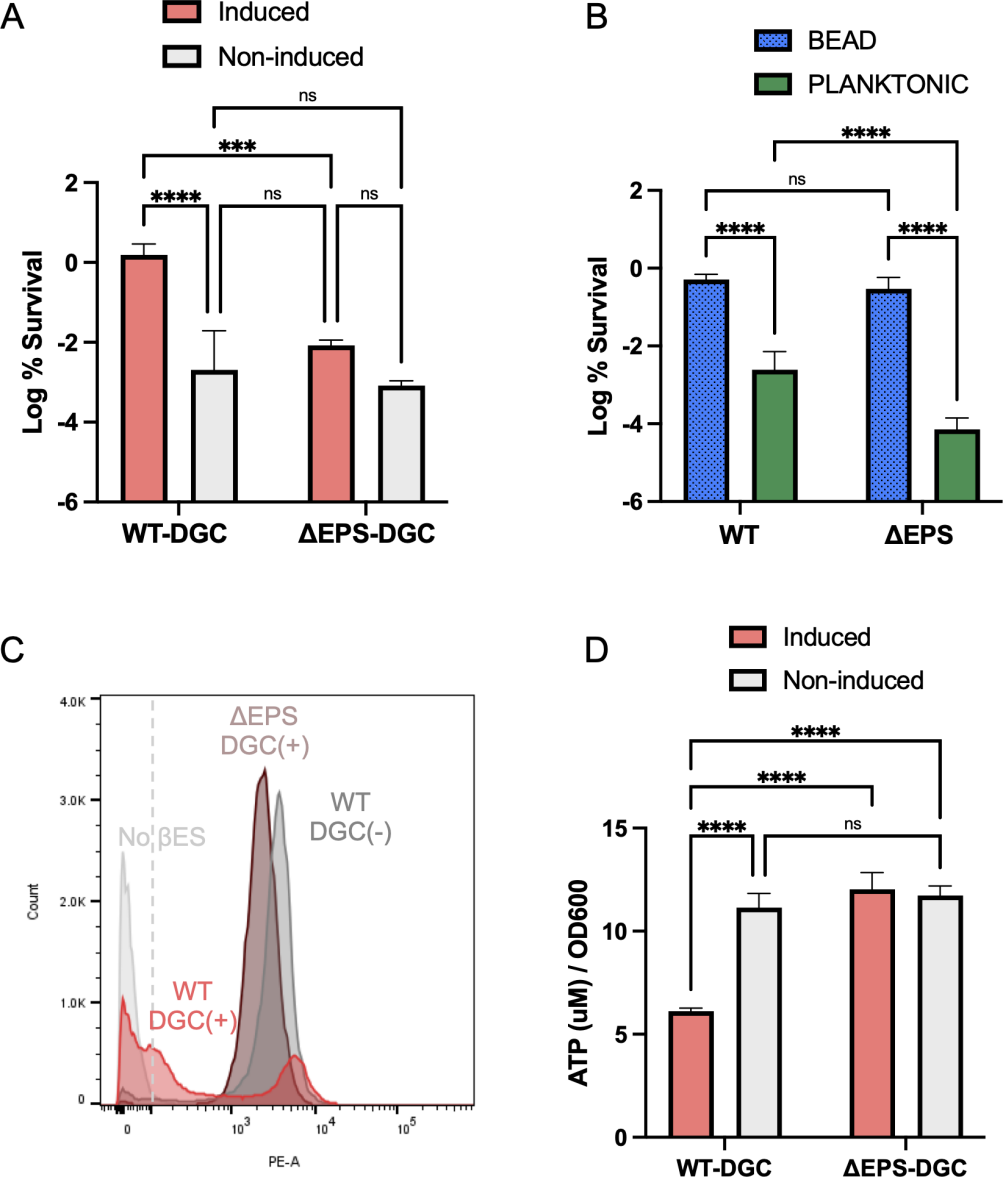
High c-di-GMP improved tolerance is dependent upon EPS production in planktonically grown cells. (**A and B**) Twenty-four hour log % survival of 10× MIC ciprofloxacin treated (**A**) PAO1 WT P_BAD_-DGC or PAO1 ΔEPS P_BAD_-DGC grown planktonically (24 h), with and without induction, (**B**) PAO1 WT and PAO1 ΔEPS grown planktonically or within alginate beads (24 h). (**C**) A 1 h βES incorporation assay to measure translation of PAO1 Δ*pvdD* P_BAD_-DGC and PAO1 Δ*pvdD* ΔEPS P_BAD_-DGC grown planktonically with inducer (red/brown) or without (gray) in stationary phase (24 h). (**D**) Bulk ATP measurement of PAO1 WT P_BAD_-DGC and PAO1 ΔEPS P_BAD_-DGC in stationary phase (24 h), with and without induction. Asterisks denote statistical significance as determined by two-way analysis of variance (ANOVA) followed by Tukey’s multiple comparisons test. ns, *P* > 0.05; ***, *P* < 0.001; ****, *P* < 0.0001.

c-di-GMP has been shown to be the major determinant of metabolic activity in *P. aeruginosa*, and much of this activity is due to increased production of EPS (47). To test whether the improvement in planktonic survival through an increase in c-di-GMP (Fig. 4A) was mediated by the metabolic demand of EPS overproduction, we moved the P_BAD_-DGC construct into a ΔEPS background (Δ*pelA* Δ*pslBCD* Δ*algD*). ΔEPS P_BAD_-DGC were grown planktonically to stationary phase (24 h) with and without inducer and challenged with antibiotic. DGC induction in the ΔEPS background did not improve tolerance as it did in WT (Fig. 5A), and the killing between the DGC induced ΔEPS was not significantly different than non-induced controls (Fig. 5A), indicating that EPS overproduction is necessary for increased survival of high c-di-GMP planktonic cells. We additionally assayed the survival of the ΔEPS strain when grown in the alginate bead. No change in killing was seen when compared to wild type (Fig. 5B); however, a 43-fold reduction in survival was seen in planktonically grown cells (Fig. 5B).

To see whether the loss of high c-di-GMP improved survival in the ΔEPS background correlates with the disappearance of a low translating population, we again measured translation rate using THRONCAT in stationary phase. As before, DGC induction and increased c-di-GMP generate a bimodal distribution in WT cells, with a population of low-translating cells seen below the nonfluorescent cutoff (Fig. 5C). However, induction of the DGC and an increase in c-di-GMP in the ΔEPS background no longer results in the emergence of a low translating population in stationary phase (Fig. 5C). Furthermore, the distribution of the induced ΔEPS P_BAD_-DGC strain is nearly identical to the non-induced WT P_BAD_-DGC (Fig. 5C), indicating that the emergence of the low translating population is dependent on the production of EPS.

As it has been shown that c-di-GMP-mediated EPS production determines metabolic activity in *P. aeruginosa* (47), we investigated whether the metabolic demand during the high c-di-GMP state resulted in depletion of available energy. We saw that maintaining a high c-di-GMP state throughout exponential growth into stationary phase (24 h) significantly depletes ATP in the bulk population (Fig. 5D), and this depletion is dependent on the ability of *P. aeruginosa* to produce EPS (Fig. 5D).

## DISCUSSION

*P. aeruginosa* is among several bacteria that successfully colonize the airways of CF patients, causing significant mortality (3). Adaptation of *Pseudomonas* to the CF lung results in chronic infection and recalcitrance to treatment by multiple classes of antibiotics (4, 5). A combination of antibiotic resistance and tolerance, including the formation of persister cells, is thought to contribute to treatment failure (48).

In this study, we explored the discrepancy between *P. aeruginosa* sensitivity to antibiotic in planktonic growth (Fig. 1A and B) with the well-established failure to eradicate *P. aeruginosa* in the clinic (3). Using THRONCAT (38), we discovered that *P. aeruginosa* is still actively translating in stationary phase when grown planktonically (Fig. 1D and E). The use of this sensitive method of non-canonical amino acid tagging (38) has revealed the otherwise obscured translational rate of stationary cells. The translational activity measured with THRONCAT correlated with the increased antibiotic sensitivity of *P. aeruginosa* (Fig. 1A and B). In contrast, *E. coli* displayed both enhanced drug tolerance (Fig. 1A and B) and considerably less translation in stationary (Fig. 1D and E). *P. aeruginosa* exhibits exceptional metabolic versatility and even in stationary phase can maintain a relatively high degree of energy production (27, 29, 49). Our findings suggest that this metabolic versatility of *P. aeruginosa* provides the energetic demands to allow for continued translation and activity of drug targets even in stationary phase, thereby increasing susceptibility to antibiotics.

The previously established alginate bead model (36) accurately replicates several key features of growth in the CF lung—embedded growth in a 3D matrix, biofilm formation, and nutrient limitation (36). We saw improved tolerance to ciprofloxacin (Fig. 2C and D) in this model and a unique bimodal distribution of translation (Fig. 3A through C). This bimodal distribution contained a population of low-translating cells unique to this bead model and not seen in planktonic cells (Fig. 3A through C), suggesting that it is members of this population that may survive the prolonged 24 h treatment with ciprofloxacin. This bimodality further suggests a threshold effect for translation under these conditions. Perhaps when ATP becomes depleted in these cells below a certain level, key bottleneck enzymes involved in translation requiring ATP stop functioning, throttling back protein synthesis.

Surface sensing and growth on solid substrates promote biofilm formation through an increase in c-di-GMP (46, 50). We tested the role of c-di-GMP and biofilm formation on antibiotic tolerance in the bead model by altering intracellular levels and saw no change in sensitivity (Fig. 4A). Our inability to alter tolerance within the bead by altering c-di-GMP is suggestive—perhaps enough stimulation for c-di-GMP production exists when *P. aeruginosa* is embedded within the beads already, making ectopic production irrelevant. Furthermore, PDE overexpression may result in a futile cycle with native DGCs, causing energy expenditure, dormancy, and subsequent drug tolerance. This will need to be explored further.

We were able to reproduce the phenomenon of increased tolerance and emergence of a low-translating population in planktonically growing cells by exogenously increasing c-di-GMP (Fig. 4A and B). This high c-di-GMP-mediated tolerance in planktonic stationary cells was further shown to be dependent on EPS production (Fig. 5A), and the loss of EPS production resulted in the disappearance of the low-translating, dormant population of cells (Fig. 5C). The clear connection between EPS production and metabolic activity (47) could explain this mechanism of tolerance, where increased metabolic demand of high c-di-GMP EPS production depletes energy in the long term, preventing *P. aeruginosa* from maintaining an active lifestyle in stationary phase (Fig. 4B and C). This shifts cells into a more dormant antibiotic-tolerant state (Fig. 4A, 5A and C). Similarly, the global regulator ProQ in *Salmonella* has been shown to activate metabolically costly flagellar and type III secretion pathways upon entrance into macrophages, inducing dormancy and improving drug tolerance (51). We have not, however, ruled out that the formation of aggregates, as happens with increased c-di-GMP even in planktonic growth and is the mode of growth in alginate beads (Fig. 2A), increases heterogeneity through limited nutrient availability, generating dormant persisters. Cell aggregation and increased metabolic demand are not mutually exclusive phenomena, and both could be contributing to increased tolerance in stationary phase.

**Fig 6:**
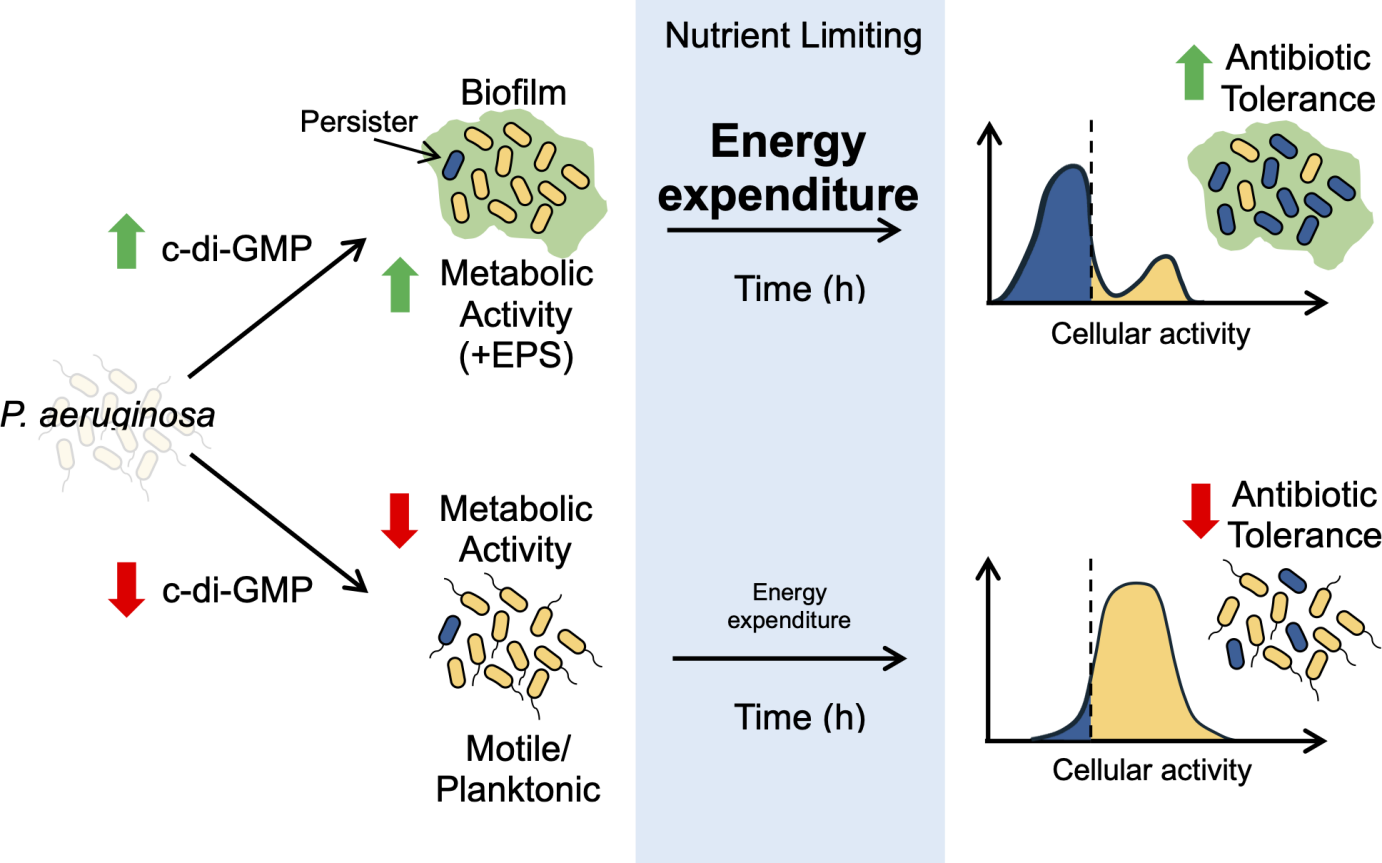
Model of persister formation in stationary phase. Growth of *P. aeruginosa* in high c-di-GMP conditions (in alginate bead, etc.) increases metabolic activity, typically through the production of EPS. Sustained activity results in greater energy expenditure over time, with a population of dormant antibiotic-tolerant persisters forming when nutrients and energy production are no longer sufficient to maintain expenditure rates.

This model (Fig. 6) of continued energy expenditure and activity describes how cells can still exhibit sensitivity to antibiotic in stationary phase (Fig. 1A, B, and D), but also how prolonged energy expenditure under nutrient-limiting conditions can generate dormant cells with drug tolerance (Fig. 2C and 4A and C). A parallel can be seen in clinical isolates of *P. aeruginosa* that display small colony variant (SCV) morphologies with reduced growth rates (52). These SCVs exhibit increased persistence in the lung (53, 54) and often contain mutations in c-di-GMP regulatory pathways (55). This contrasts with typical SCV genotypes in *S. aureus*, where reduced growth rate is the result of disruption in energy production through defects in the electron transport chain (56–58). Perhaps instead, *P. aeruginosa* generates drug-tolerant SCVs through increased dormancy from excess energy expenditure while colonizing the CF lung.

In conclusion, *P. aeruginosa* exhibits heightened antibiotic sensitivity in stationary phase because it is more translationally active (Fig. 1A, B, and D). Growth in alginate beads and an increase in c-di-GMP improve drug tolerance (Fig. 2D and 4A) and generate a population of low-translating tolerant cells (Fig. 3B and 4B and C). EPS production is implicated in this high c-di-GMP tolerance phenotype (Fig. 5A and C), and energy expenditure seems to be a determining factor of antibiotic tolerance in *P. aeruginosa*.

## SUPPLEMENTAL MATERIAL

Supplemental File mbio.03968-25-s0001.pdf Figures S1 and S2 and Tables S1 to S3.

ASM does not own the copyrights to Supplemental Material that may be linked to, or accessed through, an article. The authors have granted ASM a non-exclusive, world-wide license to publish the Supplemental Material files. Please contact the corresponding author directly for reuse.
