# Supplementary material for: Energy expenditure and cellular activity underlie antibiotic tolerance of Pseudomonas aeruginosa
Source: mBio. 2026 Feb 19;17(3):e03968-25. doi: 10.1128/mbio.03968-25 (PMC12977519; doi:10.1128/mbio.03968-25)
Supplement: Supplemental File — Figures S1 and S2 and Tables S1 to S3. [file mbio.03968-25-s0001.pdf]

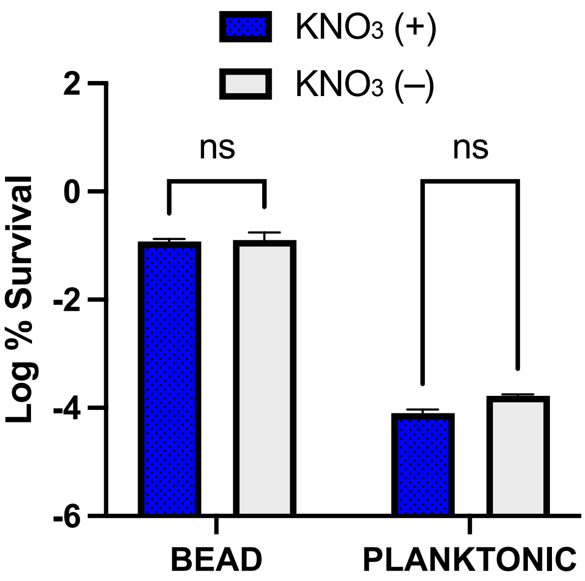

Fig. S1. No change in survival with nitrate supplementation. 24 h log % survival of 10x MIC ciprofloxacin treated WT PAO1 grown to stationary in beads and planktonic with (KNO_3 +) and without (KNO_3 –) nitrate supplementation. Asterisks denote statistical significance as determined by two-way ANOVA followed by Tukey's multiple comparisons test.

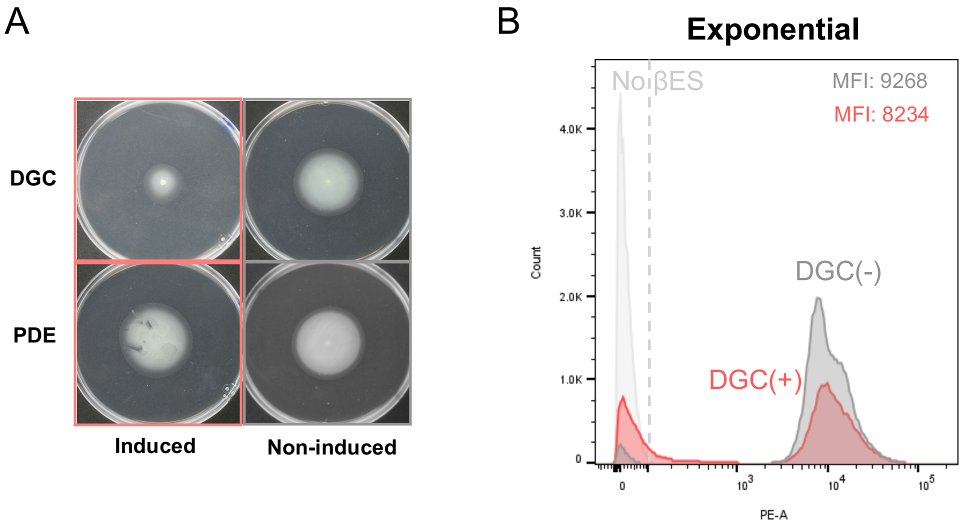

Fig. S2. c-di-GMP modulation on swimming motility and translation rate of DGC induction during exponential growth. (A) Swimming motility assay of PAO1 P_{BAD}-DGC and PAO1 P_{BAD}-PDE with and without induction. (B) THRONCAT – 1 h βES incorporation assay to measure translation of *P. aeruginosa* PAO1 Δ*pvdD* P_{BAD}-DGC grown planktonically with inducer (red) or without (grey) during exponential growth.

Table S1. Cystic fibrosis sputum media (CFSM) components and final concentrations

Component	CFSM concentration
Salmon Sperm DNA	0.6 mg/ml
Porcine Gastric Mucin Type III	5 mg/ml (prior to filter sterilization)
Glucose	3 mM
Sodium L-lactate)	9.3 mM
CaCl2·2H20	1.75 mM
MgCl2·6H20	0.606 mM
FeSO4·7H20	0.0036 mM
GlcNAc	0.3 mM
DOPC	100 μg/ml
NaH2PO4	1.3 mM
Na2HPO4	1.25 mM
KNO3	0.348 mM
K2SO4	0.271 mM
NH4Cl	2.2808 mM
KCl	14.943 mM
NaCl (adjust based on l-lactate)	51.848 mM
MOPS	10 mM
Ser	1.446 mM
Gln (CFSM uses Glu·HCl)	1.549 mM
Pro	1.661 mM
Gly	1.203 mM
Ala	1.78 mM
Val	1.117 mM
Met	0.633 mM
Ile	1.121 mM
Leu	1.609 mM
Orn·HCl	0.0676 mM
Lys·HCl	2.128 mM
Arg·HCl	0.306 mM
Trp	0.013 mM
Asp	0.827 mM
Tyr	0.802 mM
Thr	1.072 mM
Cys·HCl	0.16 mM
Phe	0.53 mM
His·HCl·H2	0.519 mM

Table S2. Effect of media and alginate beads on *P. aeruginosa* PAO1 MICs

		CFSM		MHIIB
	% Alginate	MIC (µg/ml)	MBC (µg/ml)	MIC (µg/ml)
Ciprofloxacin	2%	0.785	1.56	N/A
	1%	0.785	1.56	N/A
	0.8%	0.785	1.56	N/A
	Planktonic	0.785-1.56	1.56-3.12	0.15
Tobramycin	2%	50	50	N/A
	Planktonic	25	25	0.785-1.56

Table S3. List of strains used in this study

Designation	Genotype or Description	Source
Escherichia coli		
DH5α	Cloning strain	This work
SM10λpir	with pEX18Gm_pvdD	This work
	with pUC18T-mini-Tn7T-Gm -P_{BAD} -DGC CC3285	Kulasekara 2013
	with pUC18T-mini-Tn7T-Gm -P_{BAD} -PDE CC3396	Kulasekara 2013
MG1655	WT (CGSC #6300)	Kim 2020 (via CGSC)
Pseudomonas aeruginonsa		
PAO1	WT	This work
PAO1-iGFP	IPTG-inducible GFP; pMMB67EH-iGFP	This work
PAO1 Δ*pvdD*	Pyoverdine deletion	Kulasekara 2013
PAO1 Δ*pvdD*-DGC	Pyoverdine deletion with P_{BAD} -DGC CC3285 at attTn7 site.	This work; plasmid - Kulasekara 2013
PAO1 Δ*pvdD*-PDE	Pyoverdine deletion with P_{BAD} -PDE CC3396 at attTn7 site.	This work; plasmid - Kulasekara 2013
PAO1 ΔEPS	*ΔpelA ΔpslBCD ΔalgD* – Exopolysaccaride (EPS)-deficient	Lichtenberg 2022
PAO1 ΔEPS-DGC	EPS-deficient with P_{BAD} -DGC CC3285 at attTn7 site.	This work
PAO1 Δ*pvdD*ΔEPS-DGC	EPS-deficient and pyoverdine deletion with P_{BAD} -DGC CC3285 at attTn7 site.	This work
